# *Musa balbisiana* Seed: A Study on Extraction and the Components

**DOI:** 10.1155/ijfo/8486514

**Published:** 2026-05-26

**Authors:** Hoang Thi Ngoc Nhon, Luu Thi Quynh Hoa, Nguyen Van Anh, Le Thi Hong Anh

**Affiliations:** ^1^ Faculty of Food Science and Technology, Ho Chi Minh City University of Industry and Trade (HUIT), Ho Chi Minh City, Vietnam, huit.edu.vn

**Keywords:** MAE, *Musa balbisiana*, polyphenols, saponins, seed

## Abstract

*Musa balbisiana* seeds contain a variety of important bioactive compounds. This study evaluated the efficacy of ultrasound‐assisted extraction (UAE) and microwave‐assisted extraction (MAE) to obtain polyphenol‐ and saponin‐rich extracts from *M. balbisiana* seeds. The single‐factor influence factors, as well as the optimal conditions for the extraction, were investigated. The study also included the evaluation of the composition and content of bioactive compounds, as well as the fractionation and identification of the major compound in the purified fraction. The experimental results showed that the MAE method was superior in obtaining extracts rich in polyphenols and saponins compared to the UAE method. Under the optimal MAE conditions, the total polyphenol content (TPC) and total saponin content (TSC) in the *M. balbisiana* seed extract reached a maximum of 269.76 mgGAE/gDM and 171.36 mg/gDM, respectively, when extracted at 80% methanol, 4 s/min microwave irradiation cycle, and 40‐min microwave time. The seed extract showed characteristic peaks for polyphenols and saponins, as confirmed by Raman and FT‐IR spectra. After fractionating the seed extract, it was determined that quercetin‐3‐o‐rutinose was the major compound in the purified fraction.

## 1. Introduction

*Musa balbisiana*, also known as the wild banana, is a plant species in the *Musaceae* family from Southeast Asia that has large, hard seeds in its fruit. Polyphenols, flavonoids, tannins, and saponins are commonly found in their parts, which are secondary compounds that have potent antioxidant, anti‐inflammatory, antibacterial, hepatoprotective, and anti‐ulcer activities [1, 2]. *Musa* sp. seeds contain important fatty acids such as C16 and C18, along with ferulic acid and a significant amount of polyphenols, which contribute to the plant′s antioxidant properties, helping to neutralize free radicals and prevent chronic diseases such as cancer and diabetes [3]. *M. balbisiana* seeds showed hypoglycemic activity in vitro and in vivo as well as higher inhibitory activity against tyrosinase, xanthanoxidase, and lipase enzymes, which are involved in skin disorders, gout, and obesity, compared to other parts [4, 5]. The bioenergetic profile of free fatty acid–induced oxidative stress was improved by the bioactive fraction of *M. balbisiana* seeds, resulting in increased ATP production, respiratory, and glycolytic capacity [6]. A recent study demonstrated that the ethyl acetate fraction from *M. balbisiana* seeds exerted multidimensional protective effects against D‐galactose‐induced ageing in mice by reducing oxidative stress, inflammation, and neuronal damage, as well as improving intestinal barrier function. Furthermore, the extract had an impact on the expression of longevity‐related genes, such as SIRT1, PGC‐1*α*, and TFAM, and altered the gut microbiota in a manner that was beneficial [7]. These findings reinforce the role of *M. balbisiana* seeds as a valuable resource for applications in medicine, functional foods, and overall health. The study of improving the efficiency of obtaining its bioactive extracts is of interest to researchers.

Advanced extraction techniques, such as ultrasound‐assisted extraction (UAE) and microwave‐assisted extraction (MAE), are widely used in research to obtain extracts rich in biological activities [8]. The extraction of saponins and polyphenols from *M. balbisiana* fruits was greatly affected by the use of ultrasound and microwave waves [9]. According to the report of Putri et al., the extract from *M. balbisiana* seed obtained by UAE contains alkaloids, saponins, tannins, steroidal glycosides, and phenols. It exhibited strong antioxidant activity using DPPH and FRAP methods, with IC_50_ values ranging from 143.97 to 151.08 mg/L. The antixanthanoxidase enzyme activity was 27.95% higher than that of the control Alupurinol, showing the potential to reduce uric acid concentration [10]. The UAE method was effective in obtaining phenolics from *M. balbisiana* seeds, and the extract showed excellent antioxidant activity [11]. Microwave‐assisted aqueous (MAA) is also effective in obtaining polyphenol and tannin‐rich extracts from banana peel [12].

Several studies have been conducted on the extraction of *Musa* sp. seeds and their constituents. The efficiency of extraction methods and the analysis of bioactive extracts from *M. balbisiana* seeds have not been evaluated by any studies. In our previous study, we conducted preliminary evaluations of the effects of UAE and MAE methods on the extraction efficiency of polyphenols and saponins from *M. balbisiana* fruit, and the inhibition of xanthine oxidase in the obtained extract was determined [9]. In this study, the impacts of UAE and MAE on obtaining the extracts with high total polyphenol content (TPC) and total saponin content (TSC) from *M. balbisiana* seeds were compared, and the method with better efficiency was optimized by response surface methodology (RSM). To confirm the characteristic groups of compounds in the extract, FT‐IR and Raman spectra were employed simultaneously. By fractionating the extract and identifying the main compounds present in the seeds, the study helped to clarify the biological potential of this plant in health‐related fields.

## 2. Materials and Methods

### 2.1. Materials

*M. balbisiana* seeds were obtained from fruit harvested from the tree approximately 115–120 days after flowering in An Hoa ward (Tam Nong district, Dong Thap province, Vietnam). The seeds were first rinsed and drained in a hot‐air tray dryer (Memmert) at 60°C until they reached a moisture content of below 10%. Subsequent to drying, the material was ground to produce particles with a size of less than 80 mesh [5]. The powder was stored in a sealed bag at 4°C and used throughout all experiments. Drying at moderate temperatures (≤ 60°C) has been widely reported to be effective in reducing moisture while minimizing thermal degradation of polyphenolic compounds, thereby maintaining their stability and bioactivity [5].

The study utilized analytical‐grade chemicals and standards from Merck and Sigma‐Aldrich, including Folin–Ciocalteu reagent and gallic acid.

### 2.2. Methods

#### 2.2.1. Phytochemical Screening

The *M. balbisiana* seed powder was extracted in parallel using 80% methanol, distilled water, and 80% ethanol on separate portions of the sample, rather than through sequential extraction of the same material. The filtrates for phytochemical screening tests were obtained by filtering the mixtures through Whatman No. 1 filter paper, which identifies the major groups that comprise compounds, such as alkaloids, tannins, saponins, glycosides, steroids, anthocyanins, carotenoids, polyphenols, and flavonoids. The analysis involved color‐based reactions [13, 14]. Briefly, the bioactive compounds in the extract were identified through qualitative phytochemical tests, which were conducted based on the characteristic reactions of each compound group. For saponins, the formation of a stable foam upon addition of 0.5 N NaOH solution (pH = 13) indicated the presence of steroidal saponins, whereas the formation of a stable foam upon addition of 0.1 N HCl (pH = 1) indicated triterpenoid saponins. The confirmation of tannins was achieved by reacting with vanillin/H_2_SO_4_, resulting in deep red color characteristics. A deep yellow color was observed after reacting with a 10% NaOH solution to qualitatively identify flavonoids. The presence of alkaloids was confirmed by the appearance of a brown precipitate upon treatment with Wagner′s reagent. The Liebermann–Burchard reaction is used to recognize steroids, resulting in persistent green, pink, orange, or red colors. The Keller–Killian reaction tests for glycosides by displaying a reddish‐brown color between the two liquid layers. The identification of anthocyanins is achieved by observing the color change at different pHs: red at pH = 1 and purple at pH = 4.5. Phenolic compounds can be identified by reacting them with 5% FeCl_3_, which results in a blue‐black precipitate. Finally, the depth of yellow in the acetone extract confirms carotenoids [9, 10].

#### 2.2.2. Comparison of the Effects of MAE and UAE on Extraction From *M. balbisiana* Seed

One gram of seed powder (calculated on a dry matter basis) was extracted using the following survey methods: UAE method (ultrasonic time: 8 min, ultrasonic oscillation amplitude: 30%, and the material/solvent ratio: 1/50 w/v, using a probe‐type ultrasonic processor [Model Q500, Qsonica with the maximum power of 750 W] and MAE method (microwave irradiation cycle is 4 s/min with a power of 300 W, and the material/solvent ratio was 1/50 w/v for 30 min, using a microwave instrument: Sharp R‐370VN‐S) [15, 16]. Then, the samples were incubated at 60°C for 60 min in a thermostatic bath (WNE22 [Memmert]) before centrifugation to obtain the supernatant. The target objectives are TPC and TSC.

#### 2.2.3. Effects of MAE on Extraction From *M. balbisiana* Seed

The single‐factor experiments evaluate the individual effects of key variables, including solvent concentration, microwave power, irradiation cycle, and extraction time. Using a solid‐to‐solvent ratio of 1:30 (w/v) and a dry weight basis, 1 g of raw material (calculated on a dry weight basis) was combined with methanol at the concentrations studied (40%, 50%, 60%, 70%, and 80%). Then, the mixtures were subjected to MAE at power levels of 90, 180, 270, 360, and 540 W for extraction times of 20, 30, 40, 50, and 60 min, with microwave irradiation cycles of 2, 3, 4, and 5 s/min. An ice bath was used to cool the samples between irradiation intervals during MAE. After being treated by microwave, the extracts were incubated again in a temperature‐controlled water bath at 60°C for 60 min. The TPC and TSC were determined using UV‐Vis spectrophotometry after removing solid residues in the resulting mixtures [14].

#### 2.2.4. Optimizing Extraction Using RSM

##### 2.2.4.1. The Screening Experiment

A screening experiment (two‐level full factorial design) was performed to evaluate four variables—microwave power (W), solvent concentration (%), microwave irradiation cycle (s/min), and extraction time (min) with factor levels determined from preliminary single‐factor experiments. Extraction efficiency was assessed based on TPC and TSC. The experimental data were analyzed using Minitab 19 software to determine the relative effects of each factor and to identify those exerting significant or minor influences.

##### 2.2.4.2. The Optimizing Experiment

The experimental design used a Box–Behnken (BBK) design (RSM). Three factors at three levels (−1, 0, and +1), selected based on previous single‐factor experiments, were used to generate the experimental matrix using JMP 10 software. The independent variables included solvent concentration (%), microwave power (W), microwave irradiation cycle (s/min), and extraction time (min), whereas the response variables were TPC (Y1, mgGAE/gDM) and TSC (Y2, mg/gDM). The quadratic regression model that fully describes the system is presented in Equation (1).

(1) γ=β0+∑i=1kβiXi+∑i=1kβiiXi2+∑i=1k∑j=i+1k−1βiXiXi

In this model, *Y* represents the predicted response, whereas *β*_0_, *β*_*i*_, *β*_*ii*_, and *β*_*ij*_ denote the regression coefficients corresponding to the intercept, linear, quadratic, and interaction terms, respectively. *X*_*i*_ and *X*_*i*_ are the independent variables. The experimental data generated from the BBK design were subsequently entered into JMP 10 software for statistical analysis.

#### 2.2.5. FT‐IR, Raman, and NMR Determination

Liquid–liquid extraction using petroleum ether (PE) and an n‐butanol–water system was initially used to purify the seed extract, removing impurities like lipids and pigments. FT‐IR and Raman spectroscopy were used to characterize the resulting solution. FT‐IR spectra were recorded on a Bruker Tensor 37 spectrometer equipped with a KBr beam splitter in absorption mode over the range of 400–4000 cm^−1^ [17], whereas Raman spectra were obtained using a Cora 5X00 Raman spectrometer. The extracts were further fractionated by silica gel column chromatography employing chloroform:methanol (A), ethyl acetate:n‐hexane (B), and chloroform:n‐hexane (C) as elution solvent systems to collect fractions with higher TPC and TSC. Thin‐layer chromatography (TLC) was used to evaluate the purity of the collected fractions. The purified fraction was characterized by using a Bruker Advance DPX‐500 NMR spectrometer (Bruker, Berlin, Germany) to obtain ^1^H NMR and ^13^C NMR traces. Prior to NMR analysis, the purified sample was subjected to ultrasonic treatment at 75.5 MHz and 27°C and then dissolved in D2O at a concentration of 20 *μ*g/mL for ^1^H and ^13^C NMR measurements [18].

#### 2.2.6. Analytical Method

The Folin–Ciocalteu method was utilized to determine TPC (mg GAE/g DM) as per Feduraev et al., and the reaction mixture was tested for absorption at 765 nm [19]. TPC was calculated using the following equation: TPC (mgGAE/gDM) = *a* × *V*/*m* (Equation 2), where the calibration curve for TSC was constructed using a specified reference standard of gallic acid in the concentration range of 100–500 *μ*g/mL, *V* is the extract volume (mL), and *m* denotes the dry matter mass (g).

TSC was quantified following the method described by Chen et al. [20] and calculated using following equation: TSC (mg/gDM) = (*C*_*x*_ × *V* × *F* × 1000)/*m* (Equation 3). In this equation, *C*_*x*_ is the saponin concentration obtained from the standard calibration curve, *V* is the extract volume (mL), *F* represents the dilution factor, and *m* is the mass of dry matter in the raw material sample (g).

The colorimetric method recommended by Chlopicka et al. was employed to determine the total flavonoid content (TFC) [21]. The reaction between alkaloids and bromocresol green (BCG) was employed to quantify the total alkaloid content (TKC), and atropine was used to construct the standard calibration curve [22]. The total tannin content (TTC) was measured using the method outlined by Tambe et al. [23]. Total steroid content (TStC) was determined using the tetrazolium method in accordance with the Vietnamese Pharmacopoeia V [24]. By utilizing the pH differential method, total anthocyanin content (TAC) was compared to its original value using structural transformations of anthocyanin pigments at pH 1.0 and pH 4.5 [25]. TCC (total carotenoid content) was determined as described by Kotíková et al. [26].

### 2.3. Data Analysis

All experiments were performed in triplicate, and the results are expressed as mean ± standard deviation (SD). Statistical analyses were conducted using SPSS Statistics 20, Minitab 19, and JMP 10 software, whereas graphical representations were generated using Microsoft Excel 2019.

## 3. Results and Discussion

### 3.1. Phytochemical Screening and Quality Analysis of Bioactive Compounds

The *M. balbisiana* seed extracts were obtained using procedures described in Section 2.2.1, in the following solvents: MeOH, EtOH, and water. Subsequently, a qualitative analysis of biologically active compounds in the extract was conducted. Compound groups that produced characteristic color reactions with the applied reagents were selected for further quantitative determination. The extraction efficiencies of methanol, ethanol, and water were evaluated comparatively, and the resulting ANOVA data are summarized in Table 1.

**Table 1:** Phytochemical screening and quality analysis of bioactive compounds from *M. balbisiana* seed extract.

Groups	Phytochemical screening			Content		
	MeOH	EtOH	Aqueous	80% MeOH	80% EtOH	Water
TPC (mgGEA/gDM)	+++	+++	++	195.25 ± 1.14^a^	173.51 ± 0.38^b^	134.77 ± 0.25^c^
TSC (mg/gDM)	+++	+++	+++	107.87 ± 1.40^a^	88.68 ± 0.44^b^	55.54 ± 0.52^c^
TTC (mgGAE/gDM)	+	+	+	3.35 ± 0.17^a^	1.93 ± 0.01^b^	1.67 ± 0.03^c^
TFC (mgCE/gDM)	+++	+++	+	66.39 ± 0.49^a^	53.61 ± 0.45^b^	9.37 ± 0.09^c^
TKC (mg/gDM)	+	+	+	7.40 ± 0.21^a^	6.67 ± 0.08^b^	1.15 ± 0.41^c^
TStC (mg/gDM)	+	+	—	4.85 ± 0.04^a^	3.43 ± 0.23^b^	
TAC (mg/gDM)	+	—	—	0.011 ± 0.001	—	—
TCC (mg/gDM)	—	—	—	—	—	—

*Note:* Different letters denote statistically significant differences among solvents (MeOH, EtOH, and water) at the 5% significance level (*p* < 0.05); +, ++, and +++ indicate the relative presence of phytochemical compounds, corresponding to low, moderate, and high intensity of qualitative detection, respectively.

As shown in Table 1, methanol exhibited the highest extraction efficiency for bioactive compounds from *M. balbisiana* seeds, with a significant difference at the 5% level (*p* < 0.05). The yields of TPC, TSC, and TFC were 195.25 mgGAE/gDM, 107.87 mg/gDM, and 66.39 mg GAE/gDM, respectively. Although ethanol is suitable for food applications due to its biosafety, it only gave a medium yield. The aqueous solvent is easily available, but it has resulted in a significant level of solubility of different active groups influenced by solvent polarity, as stated in principle [27]. In addition, compounds such as anthocyanins were only present in methanol at trace levels, and carotenoids were not detected in all three solvents. Methanol was confirmed to be the most effective extraction solvent by the results and selected for further investigation experiments.

### 3.2. Comparison of the Effectiveness of MAE and UAE on Extraction

**Figure 1:**
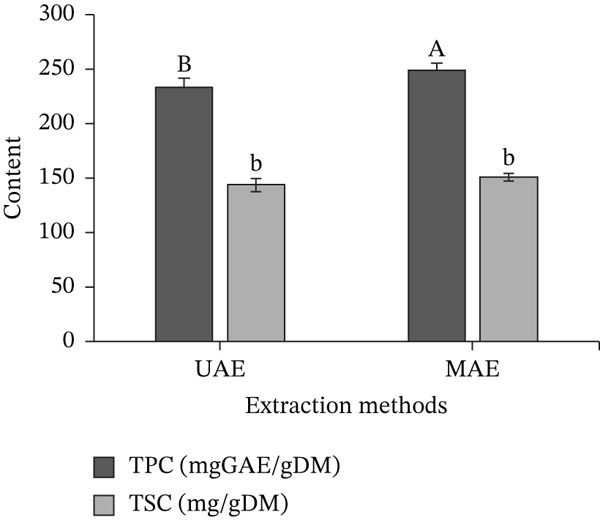
Effects of UAE and MAE methods on the polyphenols and saponins extraction efficiency from *M. balbisiana* seed. Note: In the same response, different letters on the bars indicate statistically significant differences at the 5% level (*p* < 0.05), uppercase for TPC and lowercase for TSC.

The results of the influence of UAE and MAE methods on the extraction efficiency of TPC and TSC‐rich extracts from *M. balbisiana* seed are shown in Figure 1. The results showed that the MAE method obtained extracts with higher TPC and TSC of 249.91 mgGAE/gDM and 151.19 mg/gDM than the UAE method with 234.91 mgGAE/gDM (TPC) and 144.38 mg/gDM (TSC). The superior extraction efficiency of MAE is due to the mechanical effects of internal heating caused by electrical conductivity and dielectric polarization during microwave irradiation. This process results in pressure within plant cells, which helps to disrupt cells and enhance mass transfer, while also facilitating efficient interaction between the electromagnetic field, the extraction solvent, and the plant matrix through rapid energy transfer [28].

### 3.3. Effects of the MAE Method on the Extraction of Polyphenols and Saponins From *M. balbisiana* Seeds

**Figure 2:**
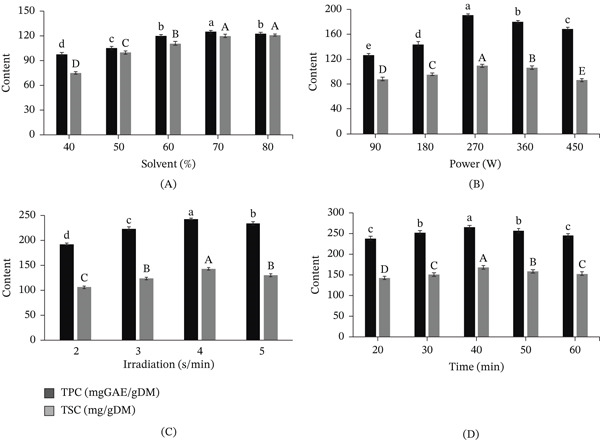
Effects of (A) solvent concentration, (B) microwave power, (C) microwave irradiation cycle, and (D) microwave time on the extraction yield of *M. balbisiana* seed extract. Note: In the same response, different letters on the bars indicate statistically significant differences at the 5% level (*p* < 0.05), uppercase for TPC and lowercase for TSC.

The efficiency of extracting polyphenol and saponin from *M. balbisiana* seeds can be influenced by solvent concentration, microwave power, irradiation cycle, and extraction time, as shown in Figure 2.

The results showed that TPC and TSC contents increased gradually with methanol concentration from 40% to 70%, reaching the highest values of 125.13 mgGAE/gDM (TPC) and 140.27 mg/gDM (TSC) at MeOH 70% (Figure 2A). As methanol concentration increased, the levels of TPC and TSC increased, which was in line with the moderate polarity of polyphenols and saponins. Methanol has been reported as a suitable solvent for extracting various phytochemicals from plants. Higher concentrations led to a slight decrease in extraction efficiency, possibly due to changes in polarity that resulted in a reduction in the solubility of hydrophilic substances, such as gallic acid and certain saponins. This trend can be attributed to the intermediate polarity of aqueous methanol, which enhances the solubility of both moderately polar phenolics and amphiphilic saponins. Similar observations have been reported, where mixed aqueous–organic solvents improve extraction efficiency by optimizing polarity and facilitating penetration into plant matrices [29]. However, a slight decrease at higher methanol concentrations (> 70%) may result from reduced solubility of more hydrophilic compounds and decreased swelling of plant tissues, limiting solvent accessibility. The effect of microwave power (Figure 2B) showed that TPC and TSC increased with microwave power from 90 to 270 W, reaching a maximum at 270 W (137.98 mgGAE/gDM and 141.31 mg/gDM), and then gradually decreased when increasing to 360 and 450 W. Moderate microwave power enhances extraction by promoting rapid internal heating, cell rupture, and improved mass transfer. However, excessive power can lead to localized overheating and degradation of thermosensitive compounds such as polyphenols and saponins. This phenomenon has been widely reported in MAE, where excessive energy input accelerates thermal degradation and oxidation reactions. Similarly, active substances are degraded at high power when extracting isoflavones from soybeans using microwaves [28]. Regarding microwave irradiation cycle (Figure 2C), the microwave irradiation cycle of 4 s/min resulted in the highest extraction with 242.91 mgGAE/gDM (TPC) and 143.32 mg/gDM (TSC). The short duration of exposure between microwaves and the sample at low irradiation cycle (2–3 s/min) did not allow for cell disruption or maximized release of active ingredients. Higher irradiation cycles are generally associated with higher recovery yield, but there is a limit. High irradiation power induces localized heating and radical formation, promoting thermal and oxidative degradation of phenolics and cleavage of saponin glycosidic bonds. Thus, the excessive frequencies could cause the reduction in yield or destruction of the active ingredient structure of compounds such as saponins or polyphenols [30]. This is consistent with previous studies that suggested that the regulation of microwave on/off cycles is an important factor in extraction [31]. The extension of the extraction time from 20 to 40 min resulted in a significant increase in both TPC and TSC, reaching a maximum of 170.35 mgGAE/gDM (TSC) and 271.46 mgGAE/gDM (TSC) (Figure 2D). However, further extension of time (50–60 min) did not increase the extraction efficiency but, on the contrary, tended to decrease, possibly due to oxidation or thermal degradation of the active ingredients. The extraction efficiency was improved by increasing the microwave time and increasing the contact between the solvent and the sample. The time was prolonged, resulting in a decrease in both TPC and TSC, which may be caused by polyphenol oxidation or thermal degradation under the influence of prolonged microwaves [32].

Accordingly, the extraction parameters for the MAE method were selected for subsequent studies, including a methanol concentration of 70%–90%, microwave power of 180–360 W, microwave irradiation cycles of 3–5 s/min, and a treatment duration of 30–60 min.

### 3.4. Optimization of Extraction by MAE From *M. balbisiana* Seed

#### 3.4.1. Screening Experiment Design

**Figure 3:**
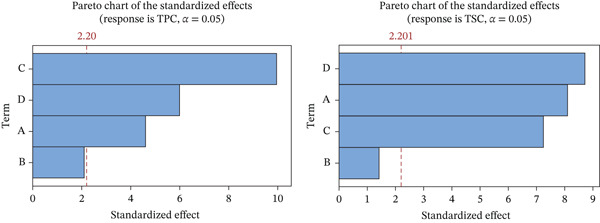
The influence of the investigated factors on TPC and TSC in *M. balbisiana* seed extract. (Factor: A: methanol concentrations, B: microwave power, C: irradiation cycle, and D: microwave time).

The purpose of the screening experiment was to identify the main and interaction effects of critical process parameters on the recovery of saponins and polyphenols from *M. balbisiana* seeds. A full factorial design at two levels was conducted, considering four independent variables: solvent concentration (*X*_1_), microwave power (*X*_2_), microwave irradiation cycle (*X*_3_), and microwave extraction duration (*X*_4_). Data were analyzed using Minitab 19. For TSC, the relative influence of the factors followed the order of microwave time > methanol concentration > irradiation cycle > microwave power. For TPC, the irradiation cycle exerted the most pronounced effect, whereas microwave power had the least impact on both TPC and TSC. The screening effects of four extraction variables on the TPC and TSC of *M. balbisiana* seed extract are summarized in Table 2. The relative significance of the investigated factors was further assessed using Pareto charts of standardized effects (Figure 3).

**Table 2:** Two‐level full factorial screening design and its effects on TPC and TSC of *M. balbisiana* seed extract.

No.	*X*_1_	*X*_2_	*X*_3_	*X*_4_	TPC	TSC
1	60	180	3	30	215.91 ± 8.74^c^	126.22 ± 3.84^e^
2	80	180	3	30	237.69 ± 9.32^bc^	144.2 ± 4.96^bcd^
3	60	360	3	30	223.13 ± 7.89^c^	127.02 ± 3.41^e^
4	80	360	3	30	236.36 ± 9.85^bc^	144.38 ± 5.12^bcd^
5	60	180	5	30	246.02 ± 10.41^abc^	139.1 ± 4.57^de^
6	80	180	5	30	243.62 ± 9.67^abc^	148.26 ± 5.68^bcd^
7	60	360	5	30	245.91 ± 10.12^abc^	140.13 ± 4.73^cde^
8	80	360	5	30	257.48 ± 11.06^ab^	157.1 ± 6.21^ab^
9	60	180	3	50	233.66 ± 8.95^bc^	139.61 ± 4.38^de^
10	80	180	3	50	244.22 ± 9.58^abc^	156.12 ± 6.04^abc^
11	60	360	3	50	237.24 ± 9.14^bc^	139.67 ± 4.62^de^
12	80	360	3	50	244.92 ± 9.76^abc^	159.2 ± 6.43^ab^
13	60	180	5	50	256.78 ± 10.88^ab^	158.59 ± 6.18^ab^
14	80	180	5	50	260.63 ± 11.21^ab^	152.66 ± 5.71^abcd^
15	60	360	5	50	258.94 ± 10.97^ab^	160.25 ± 6.59^ab^
16	80	360	5	50	269.43 ± 11.43^a^	167.79 ± 6.88^a^

The Pareto chart of standardized effects (Figure 3) further confirmed these trends, indicating that solvent concentration, irradiation cycle, and microwave time were the primary factors influencing extraction efficiency. Consequently, these three variables were selected for subsequent optimization experiments.

#### 3.4.2. Optimal Design—BBK Model

The screening experiments led to the selection of three variables for optimizing the MAE process, with TPC and TSC being the response variables. These factors were coded as *X*_1_, *X*_2_, and *X*_3_ and evaluated at three levels (−1, 0, and +1). The methanol concentration was set at 70%, 80%, and 90%, and the microwave exposure time was regulated to 3, 4, and 5 s/min, with extraction times being set at 30, 40, and 50 min. The experimental outcomes are summarized in Table 3, whereas the linear and quadratic effects of the independent variables, their interactions, and the corresponding regression coefficients were determined using JMP 10 software and are reported in Table 4.

**Table 3:** The experimental result from the optimal design RSM‐BBK.

STT	Coded			Response variables	
	*X*_1_	*X*_2_	*X*_3_	*Y*_1_ (TPC, mgGAE/DM)	*Y*_2_ (TSC, mg/gDM)
1	−1	−1	0	235.82	136.83
2	−1	1	0	244.22	140.51
3	1	−1	0	240.21	143.25
4	1	1	0	249.56	147.82
5	0	−1	−1	234.67	134.95
6	0	−1	1	245.83	147.27
7	0	1	−1	251.64	152.76
8	0	1	1	250.87	155.13
9	−1	0	−1	245.19	145.74
10	1	0	−1	257.92	153.91
11	−1	0	1	251.19	151.04
12	1	0	1	261.15	157.26
13	0	0	0	267.55	170.42
14	0	0	0	269.51	168.81
15	0	0	0	272.21	174.84

**Table 4:** Linear and square effects of independent variables, interactions between variables, and regression coefficients.

	*Y*_1_ (TPC, mgGAE/DM)	*Y*_2_ (TSC, mg/gDM)
Intercept	269.76*	171.36*
*X*_1_	4.05*	3.52*
*X*_2_	4.97*	4.24*
*X*_3_	2.45*	2.92
*X*_12_	0.24	0.22
*X*_13_	−0.69	−0.49
*X*_23_	−2.98	−2.49
*X*_11_	−9.60*	−12.40*
*X*_22_	−17.71*	−16.86*
*X*_33_	−6.29*	−6.97*
*R* ^2^	0.98	0.97
${R^{2}}_{\text{adj}}$	0.95	0.91
*F* value (model)	30.62*	17.30*
*F* value (lack of fit)	1.42	1.45

*The statistical significance is significant at the 0.05 level (*p* < 0.05).

The adequacy of the model is evidenced by the coefficient of determination (*R* ^2^), which falls within the range of 0.8–0.9, indicating a good fit [33]. The adjusted coefficient of determination (${R^{2}}_{\text{adj}}$) was also employed to further evaluate the model′s goodness of fit. It is more common to use a value that is always less than or equal to *R* ^2^ because it provides a better reflection of the fit of the multivariate linear regression model. Additionally, the model′s fit is evaluated using the *F* value of lack of fit. A good correlation model requires a good fit between the experimental results and the theory, with a lack of fit > 0.05. The analysis of variance values are necessary for the model to be considered empirical if they are significant (*p* < 0.05). From the results of Table 4, excluding the results without statistical significance, the equations have the following form:

(2) Y1=269.764.054.972.459.617.716.29+X1+X2+X3−X12−X22−X32

(3) Y2=171.363.524.2412.4016.856.97+X1+X2−X12−X22−X32.

**Figure 4:**
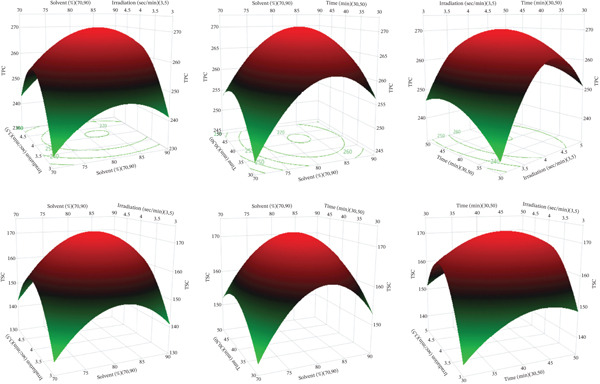
Response surface plot depicting the influence of factors on TPC and TSC.

It can be inferred from the regression models (*Y*_1_ and *Y*_2_) that most of the examined variables have measurable effects on the response functions. ANOVA performed using JMP software revealed that TPC and TSC reached 269.76 mgGAE/gDM and 171.36 mg/gDM, respectively, under the optimized conditions of 80% methanol, a microwave irradiation cycle of 4 s/min, and an extraction time of 40 min. The optimal extraction conditions can be predicted using the response surface model that illustrates the effects of the studied variables on TPC and TSC, as shown in Figure 4.

In Figure 4, it is shown that solvent concentration, microwave irradiation cycle, and extraction time are all significant factors of TPC and TSC in *M. balbisiana* seed extracts obtained by the MAE method. As the concentration of methanol increases, both TPC and TSC rise to roughly 75%–80% before declining at higher concentrations. This trend results from changes in solvent polarity, with moderate aqueous methanol improving extraction, whereas higher methanol concentrations limit the recovery of hydrophilic compounds. The extraction efficiency was initially improved by an increase in microwave irradiation cycle and extraction time because of enhanced cell disruption and diffusion. The decrease in TPC and TSC at high irradiation intensity or prolonged exposure is likely due to localized overheating and partial degradation or oxidation of bioactive compounds. The response surfaces reveal that optimal extraction happens when solvent strength, irradiation cycle, and extraction time are moderate, resulting in a balance between microwave‐enhanced mass transfer and compound stability.

At the optimal extraction conditions determined from the RSM, TPC and TSC obtained from the experiments were 249.52 ± 0.56 mgGAE/gDM and 169.38 ± 0.46 mg/gDM, respectively, compared to the corresponding predicted values of the model of 269.76 mgGAE/gDM and 171.36 mg/gDM. Although the experimental values were slightly lower than the predictions, this deviation (< 5%) was within the allowable limit, indicating that the prediction model had high accuracy and reliability in optimizing technological parameters for microwave extraction from *M. balbisiana* seed.

### 3.5. Raman and FT‐IR Spectroscopic Characterization of Preliminary Purified Extracts From *M. balbisiana* Seeds

**Figure 5:**
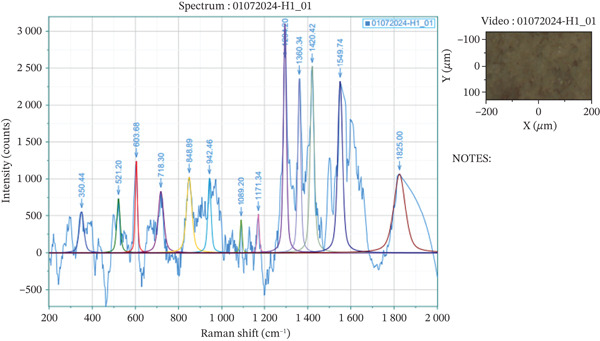
Raman spectrum of preliminary purified *M. balbisiana* seed extract.

For the preliminary purified *M. balbisiana* seed extract, the Raman spectrum exhibits vibrational bands consistent with both saponin structures and polyphenolic glycosides. In the lower spectral region, peaks at approximately 350, 521, and 604 cm^−1^ are attributed to skeletal deformations within cyclic or fused ring systems—common to triterpenoid aglycones, which form the hydrophobic core of many saponins. More critically, distinct features emerge that are closely aligned with the Raman signatures of quercetin‐3‐O‐rutinoside [34]. A peak at ~849 cm^−1^ corresponds to ring breathing vibrations of *β*‐glycosidic bonds, a hallmark of disaccharide‐linked flavonoids. Additional bands observed at 942, 1089, and 1171 cm^−1^ indicate C–C and C–O stretching in the sugar moiety (rutinose), consistent with literature reports on flavonol glycosides. Higher frequency signals, including 1294, 1360, and 1420 cm^−1^, reflect vibrations of the aromatic B‐ring and hydroxyl‐substituted phenyl groups—indicative of the quercetin scaffold. The intense band at 1549 cm^−1^ is particularly diagnostic of C=C stretching in conjugated aromatic systems, confirming the presence of flavonoid polyphenols (Figure 5).

**Figure 6:**
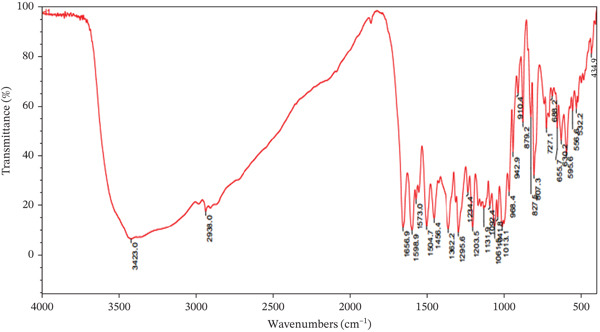
FT‐IR spectrum of preliminary purified *M. balbisiana* seed extract.

FT‐IR spectroscopy further reinforces the identification of saponins and polyphenols in the extract. A strong, broad absorption band around 3435 cm^−1^ corresponds to O–H stretching vibrations, characteristic of hydroxylated phenols and sugar groups in glycosides. Importantly, strong bands in the region of 1656–1505 cm^−1^ point to aromatic C=C and carbonyl stretching. In particular, the band at ~1656 cm^−1^ matches the conjugated carbonyl and aromatic system of the quercetin aglycone, whereas those at 1573 and 1505 cm^−1^ correspond to ring stretching vibrations of the A‐ and B‐rings in flavonoids [34]. The fingerprint region (1200–900 cm^−1^) shows multiple bands at 1203, 1131, 1092, 1033, and 968 cm^−1^ that reflect C–O–C and C–O stretching vibrations in sugar residues. These are in strong agreement with vibrational modes reported for rutinose linkages in quercetin‐3‐O‐rutinoside. Lower frequency bands at 827 and 807 cm^−1^ are attributed to aromatic C–H out‐of‐plane bending, again confirming the presence of hydroxylated flavonoid cores (Figure 6).

The combined spectral evidence from Raman and FT‐IR analyses unambiguously confirms that this extract is rich in polyphenolic compounds, with particularly strong spectral correspondence to quercetin‐3‐O‐rutinoside (rutin). The diagnostic features—such as *β*‐glycosidic vibrations, extensive C–O–C and C–O stretching, and characteristic aromatic ring deformations—match well‐established literature spectra for rutin and related flavonoid glycosides.

### 3.6. NMR Spectroscopic Characterization of Preliminary Purified Extract From *M. balbisiana* Seeds

**Figure 7:**
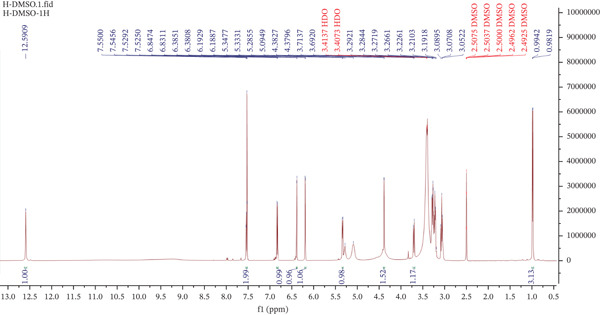
^1^H NMR spectrum of the major isolated compound from *M. balbisiana* seed extract.

For the compound called **AC** isolated from *M. balbisiana* seed extract, the NMR spectroscopic data of compound **AC** are consistent with the structural features of a flavonol glycoside. The sugar moiety is composed of two carbohydrate residues: an *α*‐L‐rhamnopyranosyl and a *β*‐D‐glucopyranosyl unit. In the ^1^H NMR spectrum (Figure 7), two meta‐coupled doublet signals are observed at *δ*_H_ 6.19 (1H, d, *J* = 2.1 Hz, H‐6) and *δ*_H_ 6.38 (1H, d, *J* = 2.1 Hz, H‐8), which are characteristic of A‐ring protons in flavonol structures. Additionally, the B‐ring displays a 1,3,4‐trisubstitution pattern, as evidenced by three aromatic proton resonances: *δ*_H_ 7.53 (1H, d, *J*_meta_ = 2.1 Hz, H‐2 ^′^), *δ*_H_ 6.84 (1H, d, *J*_ortho_ = 8.2 Hz, H‐5 ^′^), and *δ*_H_ 7.54 (1H, dd, *J*_ortho/meta_ = 8.2/2.1 Hz, H‐6 ^′^). These aromatic coupling patterns are diagnostic of the quercetin aglycone.

In the sugar region (*δ*_H_ 2.50–4.50 ppm), multiple resonances attributable to oxygenated methine protons are observed, along with two distinct anomeric proton signals at *δ*_H_ 5.34 (1H, d, *J* = 7.3 Hz, H‐1 ^″^) and *δ*_H_ 4.38 (1H, d, *J* = 1.6 Hz, H‐1 ^‴^). The larger coupling constant for H‐1 ^″^ indicates a *β*‐configuration, whereas the smaller *J* value for H‐1 ^‴^ is typical of *α*‐anomeric protons. Furthermore, a methyl doublet at *δ*_H_ 0.99 (3H, d, *J* = 6.2 Hz, H‐6 ^‴^) is indicative of the terminal methyl group of the *α*‐L‐rhamnopyranosyl residue.

**Figure 8:**
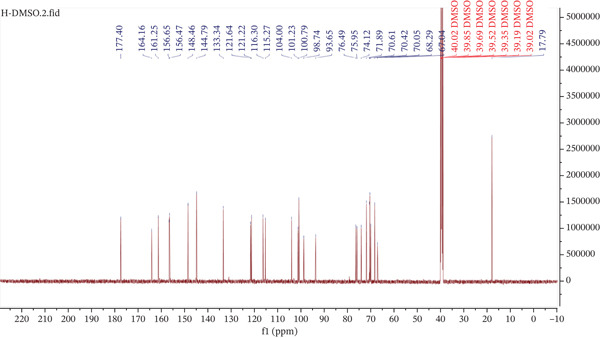
^13^C NMR spectrum of the major isolated compound from *M. balbisiana* seed extract.

**Figure 9:**
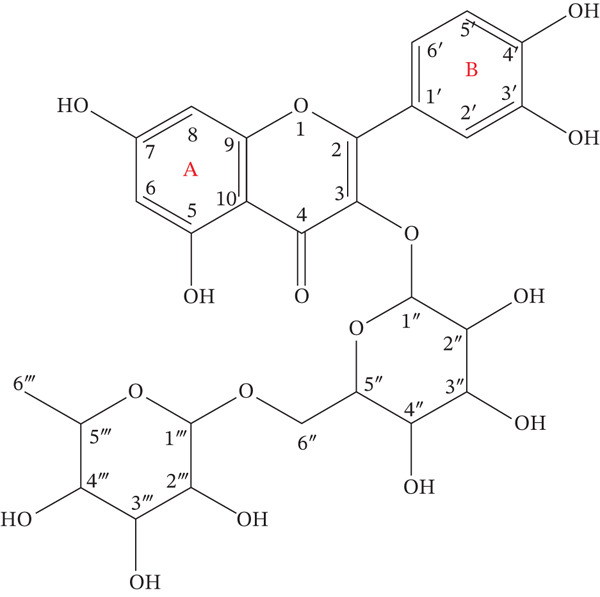
Structural elucidation of the major isolated compound from *M. balbisiana* seed extract: quercetin‐3‐O‐rutinoside.

The ^13^C NMR spectrum (Figure 8) supports the proposed structure, showing signals for all 15 carbon atoms of the flavonoid backbone. These include five aromatic CH carbons (*δ*_C_ 90.0–130.0 ppm), nine quaternary carbons (including oxygenated and nonprotonated aromatic carbons), and a carbonyl carbon at *δ*_C_ 177.4 ppm (C‐4). In the sugar region (*δ*_C_ 60.0–80.0 ppm), eight signals are assigned to oxygenated methine carbons, whereas two anomeric carbon resonances at *δ*_C_104.0 (C‐1 ^″^) and 100.7 (C‐1 ^‴^), along with a methyl carbon signal at *δ*_C_ 17.7 (C‐6 ^‴^), further confirm the presence of *β*‐D‐glucopyranosyl and *α*‐L‐rhamnopyranosyl residues, respectively. A detailed comparison of all proton and carbon chemical shifts with the documents is shown in Table 5. Based on the comprehensive analysis of both the ^1^H and ^13^C NMR spectra, compound AC is conclusively identified as quercetin‐3‐O‐rutinoside [35], commonly known as rutin (also referred to as vitamin P or osyrutin) (Figure 9).

**Table 5:** The comparison of _13_C‐NMR and _1_H‐NMR compound AC and quercetin‐3‐o‐rutinose.

Carbon no.	This work		Quercetin‐3‐o‐rutinose [35]	
	*δ* ^13^C (ppm)	*δ* ^1^H (ppm, multiplicity, *J* in Hz)	*δ* ^13^C (ppm)	*δ* ^1^H (ppm, multiplicity, *J* in Hz)
C‐2	156.89	—	156.4	—
C‐3	133.70	—	133.2	—
C‐4	177.82	—	177.3	—
C‐5	157.07	12.60 (1H, s, 5‐OH)	156.6	12.62 (1H, s, 5‐OH)
C‐6	99.15	6.20 (1H, d, *J* = 2 Hz)	98.6	6.19 (1H, d)
C‐7	164.58	—	164.0	—
C‐8	94.06	6.39 (1H, d, *J* = 2 Hz)	93.6	6.41 (1H, d)
C‐9	157.40	—	161.1	—
C‐10	104.21	—	103.9	—
C‐1 ^′^	121.41	—	121.1	—
C‐2 ^′^	115.69	7.54 (1H, d, *J* = 2.1 Hz)	115.2	7.53 (1H, d)
C‐3 ^′^	145.21	—	144.6	—
C‐4 ^′^	148.87	—	148.3	—
C‐5 ^′^	116.31	6.85 (1H, d, *J*_ortho_ = 8.2 Hz)	116.2	6.85 (1H, d)
C‐6 ^′^	121.88	7.54 (dd, *J*_ortho_ = 8.2, *J* = 2.1 Hz)	121.5	7.54 (1H, dd)
C‐1 ^″^	100.24	5.34 (1H, d, *J* = 7.3 Hz)	100.0	5.34 (1H, d)
C‐2 ^″^	73.69	3.04–3.08 (1H)	73.5	3.08 (1H)
C‐3 ^″^	75.90	3.23 (1H, d, *J* = 2.1 Hz)	75.9	3.23 (1H, d)
C‐4 ^″^	70.80	3.27 (1H)	70.8	3.27 (1H)
C‐5 ^″^	76.93	3.21 (1H, d, *J* = 2.1 Hz)	76.9	3.21 (1H, d)
C‐6 ^″^	61.27	3.73 (2H)	61.3	3.73 (2H)
C‐1 ^‴^	101.20	4.38 (1H, d, *J* = 1.6 Hz)	100.7	4.37 (1H, d)
C‐2 ^‴^	71.45	3.21 (1H)	70.9	3.21 (1H)
C‐3 ^‴^	72.00	3.36 (1H)	71.4	3.36 (1H)
C‐4 ^‴^	71.30	3.05 (1H)	70.7	3.05 (1H)
C‐5 ^‴^	72.43	3.49 (1H)	72.4	3.49 (1H)
C‐6 ^‴^	62.48	3.64 (2H)	62.3	3.64 (2H)
C‐1 ^⁗^	104.60	4.52 (1H, d, *J* = 6.2 Hz)	104.3	4.52 (1H, d)
C‐2 ^⁗^	74.07	3.31 (1H)	73.7	3.31 (1H)
C‐3 ^⁗^	76.11	3.25 (1H)	75.9	3.25 (1H)
C‐4 ^⁗^	70.87	3.29 (1H)	70.7	3.29 (1H)
C‐5 ^⁗^	81.20	3.42 (1H)	81.0	3.42 (1H)
C‐6 ^⁗^	17.70	0.98 (3H, d, *J* = 6.2 Hz)	17.6	0.97 (3H, d)

## 4. Conclusion

The findings from the study showed that the seeds of *M. balbisiana* contain a variety of bioactive phytochemicals, of which saponins and polyphenols are the two main components with the highest proportions. Quercetin‐3‐O‐rutinose was found in the seeds during the chemical analysis of the purified fraction. The study demonstrated that microwaves can be effectively utilized to aid in the extraction of TPC‐ and TSC‐enriched extracts from *M. balbisiana* seeds. Preliminary purified extracts from *M. balbisiana* seeds were analyzed by Raman and FT‐IR spectroscopy, revealing a complex phytochemical profile that was predominantly composed of saponins and polyphenols. The dominant flavonoid in the seed extract was confirmed by NMR spectroscopic data. The findings point out that *M. balbisiana* seeds are an excellent source of bioactive compounds that can be used in pharmaceutical formulations and dietary supplements. Further studies can investigate the effects of food‐grade solvents, such as ethanol or aqueous ethanol, for potential food or pharmaceutical applications in pilot‐scale or industrial settings.

## Author Contributions

Hoang Thi Ngoc Nhon led the conceptualization, investigation, and methodology, and contributed equally to manuscript review and editing. Luu Thi Quynh Hoa contributed equally to the methodology and investigation. Nguyen Van Anh contributed equally to the methodology and to manuscript review. Le Thi Hong Anh contributed equally to the conceptualization and manuscript review.

## Funding

No funding was received for this manuscript.

## Disclosure

The published manuscript version has been reviewed and approved by each author.

## Conflicts of Interest

The authors declare no conflicts of interest.

## Data Availability

The data that support the findings of this study are available from the corresponding author upon reasonable request.
